# Update: Interim Guidance for Health Care Providers for Managing Patients with Suspected E-cigarette, or Vaping, Product Use–Associated Lung Injury — United States, November 2019

**DOI:** 10.15585/mmwr.mm6846e2

**Published:** 2019-11-22

**Authors:** Tara C. Jatlaoui, Jennifer L. Wiltz, Sarah Kabbani, David A. Siegel, Ram Koppaka, Michele Montandon, Susan Hocevar Adkins, David N. Weissman, Emily H. Koumans, Michelle O’Hegarty, Megan C. O’Sullivan, Matthew D. Ritchey, Kevin Chatham-Stephens, Emily A. Kiernan, Mark Layer, Sarah Reagan-Steiner, Jaswinder K. Legha, Katherine Shealy, Brian A. King, Christopher M. Jones, Grant T. Baldwin, Dale A. Rose, Lisa J. Delaney, Peter Briss, Mary E. Evans, Lung Injury Response Clinical Working Group, Lung Injury Response Clinical Working Group, Scott Aberegg, Carolyn S. Calfee, Sean J. Callahan, Annette Esper, Anne Griffiths, Dixie Harris, Don Hayes, Devika R. Rao, Lincoln S. Smith

**Affiliations:** ^1^National Center for Chronic Disease Prevention and Health Promotion, CDC; ^2^National Center For Emerging and Zoonotic Infectious Diseases, CDC; ^3^National Center For Immunization And Respiratory Diseases, CDC; ^4^Center For Global Health, CDC; ^5^National Center for HIV/AIDS, Viral Hepatitis, STD, and TB Prevention, CDC; ^6^National Institute for Occupational Safety and Health, CDC; ^7^National Center on Birth Defects and Developmental Disabilities, CDC; ^8^Agency for Toxic Substances and Disease Registry, CDC; ^9^Emory University School of Medicine, Atlanta, Georgia; ^10^National Center for Environmental Health, CDC; ^11^National Center for Injury Prevention and Control, CDC.; University of Utah Health; Pulmonary and Critical Care Medicine; University of California; San Francisco; University of Utah; Emory University; Pediatric Pulmonary Medicine; Children’s Minnesota; Intermountain Healthcare; Nationwide Children’s Hospital; Ohio State University; Department of Pediatrics; Division of Respiratory Medicine; University of Texas Southwestern Medical Center; University of Washington; Seattle Children’s Hospital.

CDC, the Food and Drug Administration (FDA), state and local health departments, and public health and clinical stakeholders are investigating a nationwide outbreak of e-cigarette, or vaping, product use–associated lung injury (EVALI) (*1*). CDC has published recommendations for health care providers regarding EVALI (*2*–*4*). Recently, researchers from Utah and New York published proposed diagnosis and treatment algorithms for EVALI (*5*,*6*). EVALI remains a diagnosis of exclusion because, at present, no specific test or marker exists for its diagnosis, and evaluation should be guided by clinical judgment. Because patients with EVALI can experience symptoms similar to those associated with influenza or other respiratory infections (e.g., fever, cough, headache, myalgias, or fatigue), it might be difficult to differentiate EVALI from influenza or community-acquired pneumonia on initial assessment; EVALI might also co-occur with respiratory infections. This report summarizes recommendations for health care providers managing patients with suspected or known EVALI when respiratory infections such as influenza are more prevalent in the community than they have been in recent months (*7*). Recommendations include 1) asking patients with respiratory, gastrointestinal, or constitutional symptoms about the use of e-cigarette, or vaping, products; 2) evaluating those suspected to have EVALI with pulse oximetry and obtaining chest imaging, as clinically indicated; 3) considering outpatient management for clinically stable EVALI patients who meet certain criteria; 4) testing patients for influenza, particularly during influenza season, and administering antimicrobials, including antivirals, in accordance with established guidelines; 5) using caution when considering prescribing corticosteroids for outpatients, because this treatment modality has not been well studied among outpatients, and corticosteroids could worsen respiratory infections; 6) recommending evidence-based treatment strategies, including behavioral counseling, to help patients discontinue using e-cigarette, or vaping, products; and 7) emphasizing the importance of annual influenza vaccination for all persons aged ≥6 months, including patients who use e-cigarette, or vaping products.

As of November 13, 2019, 49 states, the District of Columbia, and two U.S. territories (Puerto Rico and U.S. Virgin Islands) have reported 2,172 EVALI cases to CDC, including 42 (1.9%) EVALI-associated deaths. Based on established definitions,* patients with EVALI require reported use of e-cigarette, or vaping, products within 3 months of symptom onset, positive imaging findings, and an evaluation to rule out infectious causes.

In anticipation of increasing incidence of influenza and other respiratory infections during the winter, CDC, the Council of State and Territorial Epidemiologists, state health departments, and clinical partners assessed the need for additional clinical guidance. CDC obtained individual clinical perspectives on the management of patients with suspected EVALI from nine national experts (Lung Injury Response Clinical Working Group) involved in previously published clinical guidance for EVALI patients (*4*).

## Clinical Guidance

**Patient interview.** Health care providers should ask about the use of e-cigarette, or vaping, products in a confidential and nonjudgmental manner when evaluating patients with respiratory symptoms (e.g., cough, chest pain, and shortness of breath), gastrointestinal symptoms (e.g., abdominal pain, nausea, vomiting, and diarrhea), or constitutional symptoms (e.g., fever, chills, and weight loss) (Figure). Confidentiality is essential when assessing sensitive information, including all forms of substance use, especially among adolescents and young adults.^†^ Empathetic, nonjudgmental, and private questioning of patients should be employed to encourage truthful disclosure (*8*). The most critical step in assessing EVALI is to ask patients about recent use of e-cigarette, or vaping, products. If confirmed, the types of substances used (e.g., [tetrahydrocannabinol] THC and nicotine) and where they were obtained should be ascertained. Evidence to date implicates products containing THC, particularly those obtained from informal sources like friends, family members, or in-person or online dealers (*1*,*9*). Therefore, clinicians might seek additional information to inform the ongoing investigation (Box).

**FIGURE:**
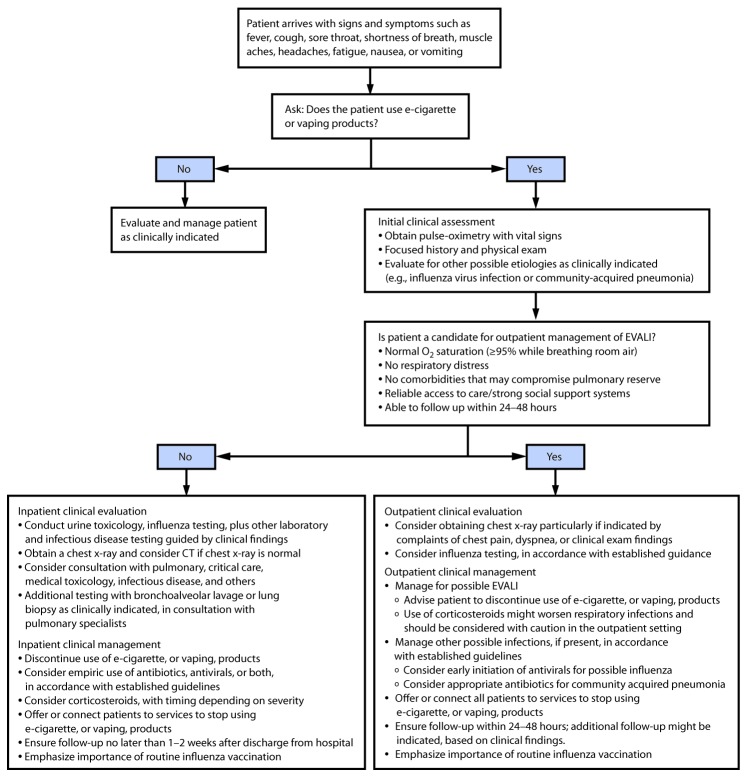
Algorithm for management of patients*^,†,§,¶^ with respiratory, gastrointestinal, or constitutional symptoms and e-cigarette, or vaping, product use **Abbreviations:** CT = computed tomography; EVALI = e-cigarette, or vaping, product use–associated lung injury. * https://www.cdc.gov/flu/professionals/diagnosis/consider-influenza-testing.htm. ^†^ https://www.cdc.gov/flu/professionals/antivirals/summary-clinicians.htm. ^§^ https://www.atsjournals.org/doi/full/10.1164/rccm.201908-1581ST#readcube-epdf. ^¶^ https://academic.oup.com/cid/article/68/6/e1/5251935.

BOX **Assessment of recent history of use of e-cigarette, or vaping products**

The most critical step in assessing e-cigarette, or vaping, product use–associated lung injury (EVALI) is to ask patients about recent use of e-cigarette, or vaping, products. Health care providers evaluating patients with respiratory symptoms (e.g., cough, chest pain, or shortness of breath), gastrointestinal symptoms (e.g., abdominal pain, nausea, vomiting, or diarrhea), or constitutional symptoms (e.g., fever, chills, or weight loss) should ask about the use of e-cigarette, or vaping, products.

- Confidentiality is essential when assessing sensitive information, including all forms of substance use, especially for young adults and adolescents.
- Empathetic, nonjudgmental, and private questioning of patients to encourage truthful disclosure should be employed.*^,^^†^
- Repeat questioning might elicit additional information about exposures, as trust is established.

The strongest evidence to date implicates products containing tetrahydrocannabinol (THC), particularly those obtained from informal sources like friends, family members, or in-person or online dealers. Therefore, it is important to ascertain the following information:

- What types of substances were used (see details below for examples)
- Where they were obtained

To assist with the ongoing investigation, the following details might provide additional necessary information:

- Types of substances used
  - THC or cannabis [specify if oil or dabs]
  - Nicotine
  - Modified products or the addition of substances (e.g., addition of vitamin E acetate)
- Product source
- Product brand and name
- Duration and frequency of use
- Time of last use
- Product delivery system
- Method of use (aerosolization, dabbing, or dripping)

* https://www.aafp.org/afp/2017/0101/p29.pdf.

^†^ https://depts.washington.edu/dbpeds/Screening%20Tools/HEADSS.pdf.

**Physical examination**. The physical exam should include assessment of vital signs and pulse oximetry; tachycardia, tachypnea, and hypoxemia have been commonly reported among cases (*4*,*9*,*10*).

**Laboratory testing and imaging studies**. Laboratory testing should be guided by clinical findings to evaluate multiple etiologies, including the possibility of EVALI and concomitant infection (*4*–*6*). A chest radiograph (CXR) should be considered for patients with a recent history of e-cigarette, or vaping, product use, who have respiratory or gastrointestinal symptoms, particularly when chest pain, dyspnea, or decreased oxygen saturation (<95% while breathing room air) are present. Measured oxygen saturation should be interpreted with consideration of factors such as altitude. A chest computed tomography scan might be considered if EVALI is in the differential diagnosis and the CXR is normal. Radiographic findings have varied and abnormalities are not present in all patients upon initial assessment (*11*). Health care providers should evaluate for causes of community-acquired pneumonia according to established guidelines as indicated by imaging findings (*12*,*13*).

**Consideration of outpatient management**. Some patients with recent history of e-cigarette, or vaping, product use who are evaluated for respiratory, gastrointestinal, or constitutional symptoms might be candidates for outpatient management. Hospital admission should be strongly considered for patients with concurrent illness such as influenza and suspected EVALI, especially if respiratory distress, comorbidities that compromise pulmonary reserve, or decreased oxygen saturation (<95% while breathing room air) are present. Candidates for outpatient management should have normal oxygen saturation (≥95%), no respiratory distress, no comorbidities that might compromise pulmonary reserve, reliable access to care, strong social support systems, and should be able to ensure follow up within 24–48 hours of initial evaluation and to seek medical care promptly if respiratory symptoms worsen; in some cases, patients who initially had mild symptoms experienced a rapid worsening of symptoms within 48 hours (*4*,*10*). Additional follow-up might be indicated, based on clinical findings.

**Influenza testing and empiric antimicrobial treatment**. Influenza testing should be strongly considered, particularly during influenza season.^§^ It might be difficult to differentiate EVALI, a diagnosis of exclusion, from influenza or community-acquired pneumonia on initial assessment, and EVALI might co-occur with respiratory infections. Treatment with empiric antimicrobials, including antivirals, should be considered in accordance with established guidelines and local microbiology and resistance patterns for bacterial pneumonia (*12*–*14*). Persons with suspected influenza who are at high risk for influenza complications, those with severe or progressive illness, and hospitalized patients are recommended for prompt administration of antiviral treatment. Antiviral treatment also can be considered for any previously healthy, symptomatic outpatient not at high risk for influenza complications, who is diagnosed with confirmed or suspected influenza, on the basis of clinical judgment, if treatment can be initiated within 48 hours of illness onset (*14*). 

**Corticosteroids and treatment of EVALI**. Corticosteroids might be helpful in treating EVALI (*4*). In published reports primarily including hospitalized patients, most patients with EVALI who received corticosteroids had rapid improvement; dosages have been previously described (*4*–*6*,*10*,*15*). In some circumstances, it would be advisable to withhold corticosteroids while evaluating patients for infectious etiologies that might worsen with corticosteroid treatment. Use of corticosteroids for the treatment of EVALI in the outpatient setting has not been well studied and should be considered with caution. Corticosteroids might worsen respiratory infections commonly seen in the outpatient setting (*13**,**14*). Some patients who have not received corticosteroids have also had clinical improvement with cessation of e-cigarette, or vaping, products (*4*–*6*,*10*,*15*), and comparative studies have not been conducted. Consultation with pulmonary, infectious disease, psychology, psychiatry, and addiction medicine specialists should be considered, as indicated, to optimize patient management.

Special consideration should be given to patients who might be at increased risk for severe outcomes with EVALI, including those who are older or have a history of cardiac or lung disease, or those who are pregnant. Among reported cases, those who were older or had past cardiac disease had more severe EVALI-associated outcomes (e.g., higher percentage requiring endotracheal intubation and mechanical ventilation and longer duration of hospitalization) (*4*).

**Discontinuation of e-cigarette, or vaping, product use**. Advising patients to discontinue use of e-cigarette, or vaping, products should be integral to the care approach. Health care providers should offer or connect patients to services to stop using e-cigarette, or vaping, products. Resuming use of these products has the potential to cause slowed recovery, recurrence of symptoms, or further lung injury (*5*). Adult patients who are using e-cigarette, or vaping, products for smoking cessation should be advised not to return to smoking cigarettes. They should be provided with evidence-based interventions, including behavioral counseling and FDA-approved cessation medications.^¶^ Adolescents and young adults might benefit from specialized services, such as addiction treatment services and providers who have experience with counseling and behavioral health follow-up. Persons with ongoing marijuana use that causes significant impairment or distress might have a cannabis use disorder. Persons with cannabis use disorder should receive evidence-based interventions such as cognitive-behavioral therapy, contingency management, motivational enhancement therapy, and multidimensional family therapy. Consultation with addiction medicine services should be considered (*16*–*18*).

**Influenza vaccination**. Health care providers should emphasize the importance of annual influenza vaccination for all persons aged ≥6 months, including their patients who use e-cigarette, or vaping products. It is not known whether patients with EVALI are at higher risk for severe complications of influenza or other respiratory infections. In addition, administration of pneumococcal vaccine should be considered for patients with a history of EVALI, according to current guidelines.**

**Postdischarge follow-up**. Patients discharged from the hospital after inpatient treatment for EVALI should have a follow-up visit within 1–2 weeks. The follow-up evaluation should include pulse-oximetry and consideration of a repeat CXR. Additional follow-up testing 1–2 months after discharge might include spirometry, diffusion capacity for carbon monoxide, and CXR.

Long-term effects and the risk for recurrence of EVALI are not known. Whereas many patients’ symptoms resolved, clinicians report that some patients have relapsed during corticosteroid tapers or with resumption of e-cigarette, or vaping, product use after hospitalization, underscoring the need for cessation and close follow-up (personal communication, Lung Injury Response Clinical Working Group, October 2019). Some patients have had persistent hypoxemia requiring home oxygen at discharge and might require ongoing pulmonary follow-up. Patients treated with high-dose corticosteroids might require care from an endocrinologist to monitor adrenal function.

Health care providers should also advise patients with a history of EVALI to return as soon as possible if they develop new or worsening respiratory symptoms, with or without fever, for early evaluation with influenza testing and early initiation of antiviral (*14*)^††^ or antibiotic treatment (*12*,*13*), as indicated.

## Public Health Recommendations

Recent testing has detected vitamin E acetate in bronchoalveolar lavage fluid samples from a convenience sample of 29 patients with EVALI (*19*); however, evidence is not yet sufficient to rule out contributions of other chemicals of potential concern contributing to EVALI. Many different substances and product sources are still under investigation, and it might be that there is more than one cause of this outbreak. Because most patients with EVALI report using THC-containing products before the onset of symptoms, CDC recommends that persons not use e-cigarette, or vaping, products that contain THC. Persons should not buy any type of e-cigarette, or vaping products, particularly those containing THC, from informal sources, like friends, family members, or in-person or online dealers.^§§^ Persons should not modify or add any substances to e-cigarette, or vaping, products that are not intended by the manufacturer; these include but are not limited to vitamin E acetate and other cutting agents and additives obtained from informal sources or purchased through retail establishments. Because the specific cause or causes of EVALI are not yet known, the only way for persons to assure that they are not at risk is to consider refraining from use of all e-cigarette, or vaping, products while the investigation continues. Irrespective of the investigation, e-cigarette, or vaping, products should never be used by youths, young adults, or pregnant women (*20*). Moreover, persons who do not currently use tobacco products should not start using e-cigarette, or vaping products. Adults using e-cigarette, or vaping, products to aid with smoking cessation should not return to smoking cigarettes; they should weigh all risks and benefits and consider using FDA-approved cessation medications.^¶¶^ Adults who continue to use e-cigarette, or vaping, products should carefully monitor themselves for symptoms and see a health care provider immediately if they develop symptoms like those reported in this outbreak.

**Summary**

**What is already known about this topic?**

A total of 2,172 U.S. e-cigarette, or vaping, product use-associated lung injury (EVALI) cases have been reported to CDC. Vitamin E acetate and tetrahydrocannabinol appear to be associated with the outbreak; however, no single causative agent has been identified.

**What is added by this report?**

As rates of influenza increase, providers evaluating patients with respiratory illnesses should ask them about e-cigarette, or vaping, product use; evaluate whether patients require hospital admission; and consider empiric use of antimicrobials, including antivirals, as well as possible corticosteroids.

**What are the implications for public health practice?**

EVALI is a diagnosis of exclusion; rapid recognition of EVALI patients by health care providers is critical to reduce severe outcomes.

## Footnotes

* https://www.cdc.gov/tobacco/basic_information/e-cigarettes/assets/2019-Lung-Injury-Surveillance-Case-Definition-508.pdf.

† https://depts.washington.edu/dbpeds/Screening%20Tools/HEADSS.pdf.

§ https://www.cdc.gov/flu/professionals/diagnosis/index.htm.

¶ https://www.cdc.gov/tobacco/campaign/tips/quit-smoking/index.html.

** https://www.cdc.gov/vaccines/hcp/acip-recs/vacc-specific/pneumo.html.

†† https://www.cdc.gov/flu/professionals/antivirals/summary-clinicians.htm.

§§
https://www.cdc.gov/tobacco/basic_information/e-cigarettes/severe-lung-disease.html#latest-outbreak-information .

¶¶ https://www.aafp.org/dam/AAFP/documents/patient_care/tobacco/pharmacologic-guide.pdf.
